# Developing social and emotional competence in higher vocational education: effects on employability and perceptions of decent work

**DOI:** 10.3389/fpsyg.2026.1786673

**Published:** 2026-05-29

**Authors:** Tongxin Zhang, Wenhua Wei

**Affiliations:** 1 Department of Physical Education, Suzhou Polytechnic University, Suzhou, Jiangsu, China; 2 School of Education, Guizhou Normal University, Guiyang, Guizhou, China

**Keywords:** China, decent work, employability, intervention study, social and emotional competence, vocational education

## Abstract

Social and emotional competence has become increasingly central in educational research due to its relevance for students’ wellbeing, adaptability, and transitions from education to work. In higher vocational education, where programs are explicitly oriented toward labor market entry, the development of social and emotional competencies may play a critical role in shaping both employability-related outcomes and students’ understanding of what constitutes decent and dignified work. However, little experimental research has examined how educational interventions targeting socioemotional competence influence vocational students’ employability perceptions and expectations regarding decent work prior to labor market entry. The present study examined the effectiveness of a six-session experiential intervention designed to foster socioemotional capacities among students enrolled in Higher Vocational Education programs in China. Using a randomized controlled experimental design, participants were assigned to an intervention group or a waiting-list control group. Quantitative data were collected at three time points (pre-test, post-test, and 4-month follow-up) to assess employability and perceptions of Decent Work Standards. A manipulation check was administered to evaluate participants’ engagement with the intervention. Results from mixed-design general linear models revealed significant Time × Group interaction effects for both employability and decent work perceptions. Students who participated in the intervention showed substantial improvements in both outcomes compared to the control group, and these gains were largely maintained at follow-up, with moderate-to-large effect sizes (η^2^ ranging from 0.09 to 0.19). These findings indicate that educational interventions targeting social and emotional competence can contribute to improving vocational students’ preparedness for the labor market and their expectations regarding fair and meaningful work. Overall, this study contributes to the emerging literature on socioemotional development in education by providing experimental evidence from a randomized design on how social and emotional competence interventions are associated with employability perceptions and expectations of decent work prior to labor market entry. By focusing on Higher Vocational Education students—a population rarely examined in intervention research—and by incorporating a longitudinal follow-up assessment, the study helps clarify how socioemotional competence development may support students’ transition from education to work. The findings highlight the importance of integrating social and emotional learning perspectives into vocational curricula and offer implications for institutions seeking to support students’ transitions into healthy, sustainable, and decent working lives.

## 1 Introduction

Social and emotional competence has become a central concern in contemporary educational research, reflecting a growing recognition that students’ successful development extends beyond academic knowledge and technical skills. Educational institutions are increasingly expected to support learners’ psychological, emotional, and interpersonal capacities, particularly in relation to wellbeing, adaptability, and participation in complex social and professional environments. Research across educational levels has consistently shown that social and emotional learning processes are associated with positive academic, psychosocial, and transitional outcomes (Lee, 2024; Körkkö and Lutovac, 2024; Zhoc et al., 2025).

Higher Vocational Education (HVE) represents a particularly important context for examining the role of socioemotional competence. Vocational programs are explicitly oriented toward preparing students for direct labor market entry and are often positioned at the intersection of educational systems and employment structures. Across many countries, vocational education systems are increasingly expected to prepare students not only with technical skills but also with the socioemotional capacities required to navigate complex and evolving labor markets. In China, HVE plays a strategic role in workforce development, serving a large student population and responding to rapid economic transformation, technological change, and evolving labor market requirements (Ministry of Education of the People’s Republic of China, 2023; Liu and Lai, 2023). Within this context, vocational institutions face the dual challenge of equipping students with occupation-specific skills while also fostering the personal and social resources required to sustain employability, wellbeing, and meaningful participation in work.

Recent research suggests that employability cannot be adequately understood as the possession of technical competencies alone. Instead, employability is increasingly conceptualized as a dynamic and multidimensional process that includes motivation, adaptability, interpersonal functioning, and self-regulatory capacities (Deng et al., 2021; Tong and Gao, 2022). Reviews of global employability frameworks highlight that social and emotional skills—such as communication, emotional regulation, teamwork, and problem-solving—are now considered core employability competencies across occupational sectors (Tushar and Sooraksa, 2023). This perspective is particularly relevant in vocational education contexts, where students often face early exposure to employment uncertainty and demanding work environments. In addition, recent research highlights the growing role of digital tools and institutional support systems in enhancing student development and employability in higher education contexts (Subbarao et al., 2025).

In parallel, growing attention has been directed toward the concept of decent work, which emphasizes not only access to employment but also the quality, dignity, and sustainability of work. Decent work encompasses safe and healthy working conditions, fair compensation, social protection, work–life balance, and alignment between individual values and organizational practices (Duffy et al., 2017; International Labour Organisation [ILO], 1999). Psychological research has shown that perceptions of decent work are closely linked to wellbeing, job satisfaction, and social inclusion (Cantos-Egea et al., 2023; Wang et al., 2024), and recent models emphasize the need to operationalize decent work as a multidimensional construct relevant to both policy and individual experience (Roša et al., 2025). Importantly, students’ expectations and representations of decent work begin to form prior to labor market entry, shaped by educational experiences and socioemotional learning processes. Vocational students may be particularly vulnerable to entering precarious or low-quality employment conditions due to their relatively early transition into the labor market and their limited bargaining power in initial job placements. From this perspective, educational interventions that shape students’ competencies and expectations can be understood as a preventive approach to promoting access to decent and dignified work.

Despite increasing recognition of the importance of social and emotional competence, relatively little research has examined how these competencies can be intentionally developed within vocational education settings and how such development may influence both employability and perceptions of decent work. Existing studies have tended to focus either on general education contexts or on employed adults, leaving a gap in understanding how socioemotional development can be fostered among vocational students as part of their preparation for work. Moreover, relatively few intervention studies in this area have employed experimental designs combined with follow-up assessments to examine whether competence-based training produces sustained changes in work-related outcomes. Addressing this gap, the present study examines the effects of a structured educational intervention designed to promote social and emotional competence among students enrolled in Higher Vocational Education programs in China.

Using an experimental design with pre-test, post-test, and follow-up assessments, this study investigates whether participation in a socioemotional competence–based intervention enhances students’ employability and their perceptions of Decent Work Standards. Vocational students represent a particularly relevant population for examining these processes, as they are approaching the transition from education to the labor market and are actively forming expectations about future working conditions and career opportunities. By focusing on vocational students—a population that remains underrepresented in international research on social and emotional competence—the study seeks to contribute to a more inclusive understanding of how educational institutions can support students’ transitions into sustainable, healthy, and decent working lives.

## 2 Theoretical background

### 2.1 Social and emotional competence in educational contexts

Socioemotional competence refers to a set of interrelated psychological and interpersonal capacities that enable individuals to understand and regulate emotions, sustain motivation, interact effectively with others, and act in accordance with personal and social values. Within educational research, these competencies are commonly discussed in relation to social and emotional learning (SEL) frameworks and include constructs such as self-awareness, emotional regulation, self-efficacy, empathy, communication skills, and ethical sensitivity. Recent scholarship emphasizes that socioemotional competence is not a peripheral educational outcome but a core dimension of teaching and learning processes (Lee, 2024; Körkkö and Lutovac, 2024). While social and emotional competence is closely related to social and emotional learning (SEL), the two concepts are not identical. SEL typically refers to structured educational processes or programs designed to foster socioemotional development, whereas social and emotional competence refers to the individual capacities and skills that such programs aim to cultivate. In the present study, socioemotional competence is conceptualized as a set of self-regulatory, interpersonal, and reflective capacities that support adaptive functioning in educational and future work contexts. This distinction is also reflected at the operational level. Social and emotional competence is assessed as an individual-level outcome through self-report measures capturing students’ perceived socioemotional capacities, whereas social and emotional learning is operationalized as the structured, experiential intervention designed to foster these competencies through guided activities, reflection, and collaborative engagement.

Social and emotional competence is also conceptually related to constructs such as social and emotional intelligence (SEI). However, the two concepts are not equivalent. Emotional intelligence typically refers to individuals’ ability to perceive, understand, and manage emotions, often conceptualized as a cognitive–affective ability or personality-related trait. In contrast, social and emotional competence is more directly amenable to educational intervention, as it encompasses skills that can be explicitly taught, practiced, and reinforced in structured learning environments. More broadly, social and emotional competence encompasses a wider set of regulatory, interpersonal, and behavioral capacities that can be intentionally developed through educational processes and applied across diverse social and institutional contexts.

A growing body of empirical evidence supports the relevance of socioemotional competence for educational engagement and student development. Meta-analytic findings indicate that teachers’ socioemotional competence is positively associated with student engagement and learning outcomes, highlighting the relational and contextual nature of socioemotional development in educational settings (Gebre et al., 2025). Studies focusing on preservice and in-service teachers further demonstrate that socioemotional capacity shapes classroom practices, professional identity, and the effective implementation of SEL-oriented curricula (Cumberledge, 2025; Sáez-Delgado et al., 2025).

Beyond teaching processes, socioemotional competence has been linked to student wellbeing, social inclusion, and positive mental health. Intervention studies show that fostering socioemotional skills can improve students’ social functioning and school wellbeing, particularly when implemented through structured, reflective, and participatory educational approaches (Hassani, 2024). Conceptual models of wellbeing in educational settings similarly emphasize the central role of socioemotional resources in supporting mental health and adaptive functioning (Frazier and Doyle Fosco, 2024).

Although much of this literature has focused on early childhood, primary, or general education contexts, recent longitudinal research suggests that socioemotional competencies developed during schooling have lasting effects on later academic and functional outcomes, including successful transitions between educational stages (Carpendale et al., 2025). These findings underscore the relevance of social and emotional competence as a developmental resource that extends beyond immediate educational contexts and into later life domains. In vocational education contexts, where students are preparing for direct entry into the labor market, these competencies may be particularly important for supporting both career-related adaptation and the development of expectations regarding work conditions.

### 2.2 Social and emotional competence and employability

Employability has increasingly been conceptualized as a dynamic process rather than a static outcome, emphasizing individuals’ capacity to obtain, maintain, and adapt employment over time (Deng et al., 2021; Tong and Gao, 2022). Contemporary employability frameworks highlight that socioemotional competencies are central to this process, supporting adaptability, resilience, interpersonal effectiveness, and self-directed career management. Reviews of global employability skills consistently identify socioemotional competencies as essential for workforce participation in the 21st century (Tushar and Sooraksa, 2023).

Empirical research conducted in educational and occupational contexts further supports this association. Studies indicate that psychological wellbeing and socioemotional resources are closely intertwined with employability trajectories, particularly during periods of labor market transition or instability (Hazelzet et al., 2020; Ke et al., 2022). Interventions that combine skill development with socioemotional training have been shown to be more effective in promoting employability than those focusing on technical competencies alone.

From a developmental perspective, social and emotional competence may influence employability through several interrelated mechanisms. Socioemotional competencies support self-regulation, goal setting, and adaptive coping, which facilitate career planning and persistence in the face of challenges. Interpersonal competencies such as communication, empathy, and collaboration also enhance individuals’ ability to navigate workplace relationships and organizational environments. Together, these capacities contribute to career adaptability and proactive career management, which are widely recognized as key components of employability development.

In vocational education systems, employability development is often considered a central educational objective, as programs are explicitly designed to prepare students for direct transitions into the labor market (Liu and Lai, 2023). Evidence from workplace settings also demonstrates the relevance of emotional and social competencies for job performance and professional functioning. For example, emotional intelligence has been identified as a critical competence influencing work performance in health-related professions (Galanis et al., 2024), and systematic reviews indicate that emotional competence training can yield positive effects across diverse occupational groups (Mehler et al., 2024). These findings reinforce the argument that employability is grounded not only in what individuals can do, but also in how they regulate emotions, interact with others, and adapt to complex social environments.

In vocational education contexts, fostering employability through social and emotional competence development may be particularly important. Vocational students often face early entry into demanding work environments and may have limited opportunities for gradual professional socialization. Supporting socioemotional development during vocational training may therefore enhance students’ confidence, adaptability, and readiness to engage with evolving labor market demands.

### 2.3 Social and emotional competence and decent work

Beyond employability, socioemotional competence is also closely related to individuals’ perceptions and experiences of work quality. The concept of decent work emphasizes not only employment access but also dignity, fairness, safety, and sustainability in working conditions (International Labour Organisation [ILO], 1999). Psychological research grounded in the Psychology of Working Theory has demonstrated that access to decent work is strongly associated with wellbeing, mental health, and social inclusion (Duffy et al., 2017; Ma et al., 2023).

Perceptions of decent work are shaped by values, expectations, and interpretations of workplace conditions—processes that are inherently socioemotional. Empirical studies show that workers’ perceptions of fairness, value alignment, and psychological safety are linked to emotional wellbeing and satisfaction (Cantos-Egea et al., 2023; Wang et al., 2024). Recent efforts to model and evaluate decent work further emphasize its multidimensional and experience-based nature, highlighting the relevance of psychological and social factors alongside structural indicators (Roša et al., 2025).

Importantly, expectations regarding decent work begin to develop prior to labor market entry, during the educational period in which students form initial representations of work quality, rights, and acceptable working conditions. Educational environments play a formative role in shaping how students conceptualize work quality, rights, and responsibilities. From this perspective, fostering social and emotional competence within education may influence not only employability outcomes but also students’ aspirations toward fair, meaningful, and dignified work. Socioemotional competencies such as self-awareness, ethical sensitivity, and interpersonal responsibility may support students in evaluating potential work environments and recognizing the importance of fairness, safety, and value alignment in future employment. Examining these processes among vocational students is particularly relevant, as they are approaching the transition from education to work and are actively forming expectations about employment quality before entering the labor market.

### 2.4 From theory to intervention in higher vocational education

Integrating socioemotional competence development into vocational education requires intentional pedagogical design grounded in theory. Human Capital Theory emphasizes investment in competencies that enhance productivity and employability (Nafukho et al., 2004), while Self-Determination Theory highlights the role of competence, autonomy, and relatedness in fostering motivation, engagement, and wellbeing (Gagné and Deci, 2005). Complementing these perspectives, the Capability Approach emphasizes agency and the expansion of individuals’ real opportunities to pursue work they value (Hart, 2012; Robeyns, 2021).

Drawing on these frameworks, the intervention examined in the present study was designed to foster socioemotional capacities relevant to vocational students’ transition into work, including self-efficacy, motivation, interpersonal communication, emotional regulation, resilience, reflective career planning, and awareness of decent work principles. The intervention was structured into six sessions in order to provide sufficient time for progressive development of socioemotional competencies through experiential activities, reflection, and group discussion while remaining feasible within the academic schedules of vocational education programs. Rather than focusing exclusively on technical training, the program emphasized experiential learning, collaborative activities, and critical reflection, aligning educational practices with students’ psychological and social development needs. This approach is consistent with recent research highlighting the value of experiential and technology-supported learning environments in vocational education for enhancing students’ competencies, motivation, and engagement (Luptáková et al., 2025; Shannaq et al., 2025).

Each theoretical perspective informed specific elements of the intervention design. Human Capital Theory guided the focus on competencies that strengthen students’ long-term employability and adaptability in the labor market. Self-Determination Theory informed activities aimed at supporting motivation, self-efficacy, and collaborative learning through participatory and reflective exercises. The Capability Approach provided a broader normative framework emphasizing students’ agency and their capacity to envision and pursue forms of work aligned with dignity, wellbeing, and decent work principles. Rather than functioning as independent perspectives, these frameworks are conceptually integrated within the intervention design. Human Capital Theory provides the foundational rationale for investing in competencies linked to employability outcomes, Self-Determination Theory explains the motivational processes through which these competencies are developed and internalized, and the Capability Approach extends this logic by emphasizing how such competencies expand students’ perceived agency and opportunities to pursue meaningful and dignified work. Together, these theoretical perspectives provide a mechanism through which socioemotional competence development may strengthen students’ motivation, agency, and interpersonal capacities, thereby supporting employability development and shaping expectations regarding decent work.

By situating the intervention within this integrated theoretical framework, the present study contributes to a more nuanced understanding of how social and emotional competence can be developed in vocational education and how such development may influence employability and perceptions of decent work prior to labor market entry.

#### 2.4.1 Hypotheses

Drawing on the literature reviewed above, the present study tested four hypotheses examining the effects of a socioemotional capacities–based intervention on vocational students’ employability and perceptions of decent work.

H1: Participation in the social and emotional competence–based intervention will significantly increase students’ employability compared with a control group.

H2: Participation in the intervention will lead to significantly more positive perceptions of Decent Work Standards compared with a control group.

H3: Within the intervention group, the increase in employability observed after the intervention will be maintained over time at follow-up.

H4: Within the intervention group, the improvement in perceptions of Decent Work Standards observed after the intervention will persist at follow-up.

Overall, this study seeks to contribute to both theory and practice by empirically assessing how structured educational interventions targeting socioemotional competence can promote employability and shape work-related values among students in Higher Vocational Education. Because the primary objective of the study was to evaluate the effectiveness of the intervention on employability and perceptions of Decent Work Standards over time, the hypotheses focused on direct intervention effects. Examining potential mediating mechanisms linking socioemotional competence development to these outcomes represents an important direction for future research but was beyond the scope of the present design. By focusing on a population that remains underrepresented in international research on social and emotional competence, the study provides practical insights for the design of institutional strategies that support students’ transition into work and foster sustainable, healthy, and decent working lives.

The figure presents a schematic representation of the theoretical model underlying the intervention and the hypotheses examined in the study. The upper panel illustrates the proposed intervention mechanism, whereby experiential learning, collaborative activities, and critical reflection are expected to contribute to the development of social and emotional competence. The middle panels depict the expected between-group effects tested in Hypotheses 1 and 2, showing higher levels of employability and more positive perceptions of Decent Work Standards in the intervention group compared with the control group at post-test. The lower panels illustrate the longitudinal trajectories examined in Hypotheses 3 and 4, indicating the maintenance of post-intervention gains in employability and perceptions of Decent Work Standards at follow-up within the intervention group. All graphical elements are intended to illustrate the hypothesized relationships and patterns of change and do not represent exact empirical values or scale metrics.

## 3 Materials and methods

### 3.1 Participants

One hundred thirty-nine students enrolled in Higher Vocational Education (HVE) institutions in China participated in this study. Participants were drawn from two groups: an experimental group (*n* = 79) that received the social and emotional competence–based intervention, and a control group (*n* = 60) placed on a waiting list. Participants’ ages ranged from 18 to 22 years, which is typical for the Chinese HVE population, where students generally begin higher vocational studies around the age of 18 and graduate by approximately 21 years old. The mean age of the experimental group was 19.47 years (SD = 1.16), while the control group had a slightly higher mean age of 20.02 years (SD = 1.08).

Regarding gender distribution, the experimental group included 34 male and 45 female students, whereas the control group consisted of 31 male and 29 female students, resulting in a relatively balanced overall gender composition (47% male, 53% female). All participants were full-time students enrolled in higher vocational institutions. There were no missing data for age or gender variables across groups.

### 3.2 Procedure

The study protocol, including participant recruitment, consent procedures, and the data management plan, was reviewed and approved by the Institutional Ethics Committee of Guizhou Normal University (Approval No. EDU-GZNU–20232203, approved on November 5th, 2023). All research activities adhered to the ethical principles of the Declaration of Helsinki (2013) and the American Psychological Association’s Ethical Standards for Research with Human Participants (American Psychological Association [APA], 2017). Although the study was not preregistered prior to data collection, the experimental protocol was retrospectively registered in the Open Science Framework (OSF) on November 3rd, 2025, in accordance with transparency guidelines for human intervention studies.

All participants received a written and verbal explanation of the study’s objectives, procedures, and voluntary nature during the first week of March 2024, prior to the start of data collection. They were informed of their right to withdraw at any time without academic or personal consequences. Written informed consent was obtained individually between March 4th and March 8th, 2024, immediately before the administration of the pre-test measures. Confidentiality and anonymity were ensured by assigning identification codes instead of personal identifiers, and all data were stored on password-protected university servers accessible only to the research team.

The study was implemented through the Jiangsu–Guizhou Vocational Education Research Consortium (JGVERC), a collaborative network coordinated by Guizhou Normal University in partnership with Suzhou Polytechnic University, Jiangsu Vocational Institute of Commerce, and Nanjing Industrial Vocational College. These partner institutions jointly supported participant recruitment, local coordination, and the implementation of the intervention within their respective Higher Vocational Education (HVE) programs. These institutions were selected because they are active members of the consortium and regularly collaborate in joint research and educational innovation initiatives within the Higher Vocational Education system. Their participation ensured access to comparable student populations and the logistical capacity to host the intervention and data collection procedures.

A total of 162 students initially expressed interest in participating in the study. Following eligibility screening, 23 students were excluded for not meeting inclusion criteria (e.g., not being enrolled in full-time HVE programs) or for declining to complete the informed consent procedures. The final sample consisted of 139 students, who were randomly assigned to either the experimental group (*n* = 79) or the control group (*n* = 60). Group allocation was performed using a computerized randomization procedure by a research team member who was not involved in the delivery of the intervention or the data collection process, in order to minimize potential allocation bias. Although full allocation concealment could not be guaranteed due to the educational setting, the use of computerized randomization and the separation between assignment and intervention delivery procedures substantially reduced the likelihood of systematic selection bias.

Sample size estimation was conducted using G*Power 3.1 software. The analysis indicated that a minimum of 128 participants would be required to detect a medium effect size (f = 0.25) with 95% statistical power at a significance level of 0.05 for a repeated-measures ANOVA involving two groups across two time points. The final sample exceeded this threshold, ensuring adequate statistical power for hypothesis testing.

At Time 1 (pre-test), conducted between March 6th and March 8th, 2024, participants in both groups completed self-report measures assessing employability and perceptions of Decent Work Standards. Data collection took place in classroom facilities at Suzhou Polytechnic University (Suzhou, Jiangsu Province) and Guizhou Normal University (Guiyang, Guizhou Province), the two primary research sites within the consortium. Surveys were administered in supervised group sessions led by trained research assistants who were not involved in teaching activities, in order to minimize potential response bias.

Following the pre-test, participants in the experimental group took part in a structured social and emotional competence–based intervention delivered in person at the vocational training center of Suzhou Polytechnic University. The intervention consisted of 6 weekly sessions conducted between March 11th and April 19th, 2024, with each session lasting approximately 2 h. The program was designed to foster core social and emotional competencies relevant to students’ transition into work, including self-efficacy, motivation, emotional regulation, interpersonal communication, career adaptability, resilience, and awareness of decent work principles. Grounded in experiential learning and socio-constructivist approaches, the intervention emphasized reflective activities, collaborative learning, and guided discussions linking personal development with work-related values and expectations. For example, activities included guided role-playing scenarios focused on workplace communication and conflict management, reflective exercises aimed at identifying personal strengths and career goals, and small-group discussions addressing ethical dilemmas and expectations related to decent work. A detailed description of the intervention content and structure is provided in Supplementary Appendix 1.

Each session was facilitated by two certified educators affiliated with the Department of Physical Education and the School of Education, respectively. Both facilitators had received prior methodological training on intervention delivery, ethical conduct, and participant protection. Sessions were held in the “Innovation and Employability Development Center” at Suzhou Polytechnic University, which offers dedicated spaces for interactive workshops and small-group activities. To ensure consistency in delivery, both facilitators followed a standardized session protocol and received joint training prior to implementation.

Participants in the control group were placed on a waiting list and did not participate in interactive sessions during the intervention period. Instead, they received informational reading materials related to employability and decent work, distributed digitally via the university’s learning management system.

At Time 2 (post-test), conducted between May 2nd and May 6th, 2024, participants in both groups completed the outcome measures again. In addition, a manipulation check was administered to participants in the experimental group to assess engagement with the intervention and perceived relevance of the activities for their socioemotional and work-related development. Data collection procedures mirrored those used at pre-test and were conducted under standardized conditions by the same independent research assistants.

At Time 3 (follow-up), approximately 4 months later—between September 2nd and September 6th, 2024, coinciding with the beginning of the new academic term—participants in the experimental group completed a follow-up assessment to evaluate the sustainability of changes in employability and perceptions of Decent Work Standards. The control group did not participate in the follow-up assessment but was subsequently granted full access to the intervention materials and attended a debriefing session outlining the study’s aims, outcomes, and the ethical rationale underlying the waiting-list design.

The procedural flow was designed to minimize disruption to academic schedules while allowing for a rigorous evaluation of both short- and longer-term effects of the intervention. All intervention activities and data collection procedures were conducted under the oversight of the Jiangsu–Guizhou Vocational Education Research Consortium and monitored by institutional representatives to ensure compliance with ethical and procedural standards. To promote transparency and reproducibility, anonymized datasets, informed consent materials, the full experimental protocol, and a detailed description of the intervention have been deposited in an Open Science Framework repository. These materials can be accessed at the following link: https://osf.io/vdafz/overview?view_only=f7a528fb331646e5a6b4826e1da3983b.

### 3.3 Instruments

#### 3.3.1 Employability

Students’ employability was assessed using the Future Employability Scale, which measures individuals’ anticipated ability to obtain and sustain employment and to foster future career development based on current self-perceptions. This self-report instrument was specifically developed for Chinese college students and demonstrates strong psychometric properties (Deng et al., 2021). The scale is grounded in the conservation of resources theory (Hobfoll, 2001), the possible selves framework (Cross and Markus, 1991), and social positioning perspectives (Lawson, 2022, 2025), and adopts an output-oriented approach to employability assessment.

The scale consists of 28 items distributed across four dimensions. Knowledge and Skills (Items 1–7) assesses perceived possession of core professional, technical, and general workplace competencies. Personality Quality (Items 8–14) captures socioemotional attributes relevant to future work roles, such as enthusiasm, responsibility, ethical orientation, and emotional regulation. Interpersonal Network (Items 15–21) evaluates individuals’ perceived ability to establish, maintain, and mobilize professional relationships in career contexts. Career Development (Items 22–28) assesses perceived readiness for labor market transitions, long-term competitiveness, and career adaptability. Items are rated on a 5-point Likert scale ranging from 1 (strongly disagree) to 5 (strongly agree), with higher scores indicating greater perceived future employability. As a self-report measure, the scale captures students’ subjective perceptions of their future employability, which are theoretically meaningful in the context of educational transitions. Perceived employability has been shown to function as an important psychological resource influencing motivation, career behavior, and adaptation to labor market demands. Although self-reported measures may be subject to response biases, they are widely used in employability research and are particularly appropriate when examining anticipatory constructs prior to actual labor market entry.

The scale has demonstrated excellent internal consistency (Cronbach’s α = 0.974), split-half reliability (0.925), and test–retest reliability (0.912). Construct validity has been supported through confirmatory factor analysis, and criterion validity has been established via associations with perceived employability, university commitment, and academic satisfaction (Chen et al., 2023).

#### 3.3.2 Decent work standards

Perceptions of decent work were assessed using the Chinese version of the Decent Work Scale (C-DWS), originally developed by Duffy et al. (2017) and subsequently adapted and validated for Chinese populations by Ma et al. (2023). The C-DWS comprises 15 items grouped into five subscales: (1) physically and interpersonally safe working conditions (e.g., “I feel emotionally safe interacting with people at work”), (2) access to healthcare (e.g., “I get good healthcare benefits from my job”), (3) adequate compensation (e.g., “I am not properly paid for my work,” reverse scored), (4) time and rest (e.g., “I do not have enough time for non-work activities,” reverse scored), and (5) organizational values match (e.g., “The values of my organization match my family values”).

Items are rated on a 7-point Likert scale ranging from 1 (strongly disagree) to 7 (strongly agree). In the original validation study, internal consistency reliabilities ranged from α = 0.77 to 0.87. The Chinese validation reported Cronbach’s α values between 0.68 (safety) and 0.87 (compensation), indicating acceptable to high reliability. Factorial analyses supported the use of both subscale and total scores, with high overall reliability (ω = 0.93), as well as measurement invariance across gender and work sectors and good convergent and predictive validity (Ma et al., 2023). Although the Decent Work Scale was originally developed for individuals with work experience, it has also been used to examine perceptions and expectations of work quality among individuals preparing to enter the labor market. In the present study, participants were instructed to respond to the items in relation to anticipated or future employment conditions rather than current full-time work experiences. This approach is consistent with research examining work-related expectations during the education-to-work transition. These anticipatory perceptions are theoretically meaningful, as expectations regarding work quality are known to shape career-related motivation, decision-making, and future adjustment to employment conditions. Assessing anticipated decent work therefore provides a valid approach to capturing how students evaluate and internalize standards of work quality prior to labor market entry.

#### 3.3.3 Manipulation check

To assess participants’ engagement with and perceived relevance of the intervention, a manipulation check was administered to the experimental group immediately following the post-test assessment. This self-report questionnaire consisted of five items developed specifically for this study to evaluate cognitive, affective, and perceived developmental responses to the social and emotional competence–based intervention. Items were rated on a 5-point Likert scale ranging from 1 (strongly disagree) to 5 (strongly agree).

The five items assessed whether participants found the sessions engaging, perceived the content as relevant to their future career goals, believed the intervention contributed to their employability-related development, gained new perspectives on decent work, and would recommend the program to other vocational students. These items served as an internal validity check to ensure meaningful engagement with the intervention content and to verify that the program was perceived as relevant to students’ socioemotional and work-related development.

### 3.4 Data analyses

All statistical analyses were conducted using IBM SPSS Statistics, version 28. Prior to hypothesis testing, descriptive statistics and Pearson bivariate correlations were computed to examine central tendencies and associations among the main study variables, including employability and perceptions of Decent Work Standards. Prior to conducting the main analyses, the assumptions of the General Linear Model were examined, including normality, absence of extreme outliers, and, where applicable, sphericity. Inspection of skewness and kurtosis values and graphical diagnostics indicated no substantial departures from normality, and no extreme outliers were detected. No missing data were observed in the dataset.

To test the study hypotheses, a series of mixed-design General Linear Models (GLMs) were conducted to evaluate the effectiveness of the social and emotional competence–based intervention. In these models, Time (pre-test, post-test, and, when applicable, follow-up) was specified as a within-subjects factor, and Group (experimental vs. control) was specified as a between-subjects factor. The primary focus was on the Time × Group interaction effects.

For Hypothesis 1, a 2 (Time: pre-test vs. post-test) × 2 (Group: experimental vs. control) repeated-measures GLM was performed on employability scores. For Hypothesis 2, an analogous model was applied to perceptions of Decent Work Standards. To examine Hypotheses 3 and 4, which addressed the maintenance of intervention effects over time, three-level within-subjects GLMs (pre-test, post-test, follow-up) were conducted within the experimental group only, focusing on changes in employability and perceptions of Decent Work Standards across measurement occasions.

Multivariate test statistics (Pillai’s Trace and Wilks’ Lambda) were examined to assess the robustness of the models, and Mauchly’s Test of Sphericity was used to evaluate the assumption of sphericity. When violations of sphericity were detected, Greenhouse–Geisser corrections were applied. Effect sizes were reported using partial eta squared (η^2^), and estimated marginal means were computed to facilitate the interpretation of significant interaction effects. For all analyses, statistical significance was set at *p* < 0.05.

## 4 Results

### 4.1 Descriptive and correlational analyses

Descriptive statistics for all study variables are presented in Table 1. At baseline, participants reported moderate to high levels of employability (*M* = 3.11, SD = 0.65), with higher mean levels observed at post-intervention (*M* = 3.41, SD = 0.76) and at follow-up (*M* = 3.63, SD = 0.49). Similarly, perceptions of Decent Work Standards were moderate at baseline (*M* = 2.51, SD = 0.57), increased following the intervention (*M* = 2.77, SD = 0.73), and remained elevated at follow-up (*M* = 2.84, SD = 0.62).

**TABLE 1:** Descriptive statistics for key variables and Pearson correlations.

Variable	M	SD	1	2	3	4	5
1. Employability (pre)	3.11	0.65	—	—	—	—	—
2. Employability (post)	3.41	0.76	0.622**				
3. Employability (follow)	3.63	0.49	0.307**	0.595**			
4. Decent work (pre)	2.51	0.57	0.502**	0.455**	0.211		
5. Decent work (post)	2.77	0.73	0.270**	0.396**	0.108	0.544**	
6. Decent work (follow)	2.84	0.62	0.186	0.221	0.278*	0.427**	0.856**

*p < 0.01,

**p < 0.05. Pearson’s r reported.

Pearson correlation analyses were conducted to examine associations between employability and perceptions of Decent Work Standards across the three measurement occasions. As shown in Table 1, baseline employability was significantly correlated with both post-intervention and follow-up employability scores. In addition, post-intervention employability was strongly associated with follow-up employability, indicating temporal consistency in participants’ perceptions of their employability over time.

A similar pattern was observed for perceptions of Decent Work Standards. Baseline Decent Work perceptions were significantly correlated with post-intervention and follow-up scores, and post-intervention Decent Work perceptions were strongly related to follow-up scores, further supporting the stability of perceived work standards across measurement points.

Cross-construct correlations revealed significant positive associations between employability and perceptions of Decent Work Standards at all three time points. For example, baseline employability was positively correlated with baseline and post-intervention Decent Work perceptions. Notably, employability and perceptions of Decent Work were positively associated at post-test, suggesting a convergence between students’ perceived career readiness and their expectations regarding work quality. However, the strength of these associations was generally attenuated at follow-up, particularly for the correlation between employability and Decent Work at follow-up. This pattern suggests that although employability and perceptions of Decent Work are related constructs, they may follow partially distinct developmental trajectories over time.

### 4.2 Employability

To examine the effects of the social and emotional competence–based intervention on employability, a mixed-design repeated-measures ANOVA was conducted, with Time (pre-test vs. post-test) as a within-subjects factor and Group (experimental vs. control) as a between-subjects factor. The analysis included 139 participants, with 79 students in the experimental group and 60 students in the control group.

Descriptive statistics indicated that, at baseline, the experimental group reported a mean employability score of *M* = 3.1060 (SD = 0.6496), while the control group reported a comparable mean score of *M* = 3.1125 (SD = 0.6488). At post-test, the experimental group showed a marked increase in employability (*M* = 3.6424, SD = 0.5745), whereas employability levels in the control group remained relatively stable (*M* = 3.1000, SD = 0.8647). Independent-samples t-tests were conducted to examine baseline equivalence between the experimental and control groups. Results indicated that the groups did not differ significantly in employability at pre-test, *t*(137) = -0.058, *p* = 0.954, nor in perceptions of Decent Work Standards, *t*(137) = -0.775, *p* = 0.440. These results suggest that the two groups were comparable at baseline prior to the intervention.

**FIGURE 1:**
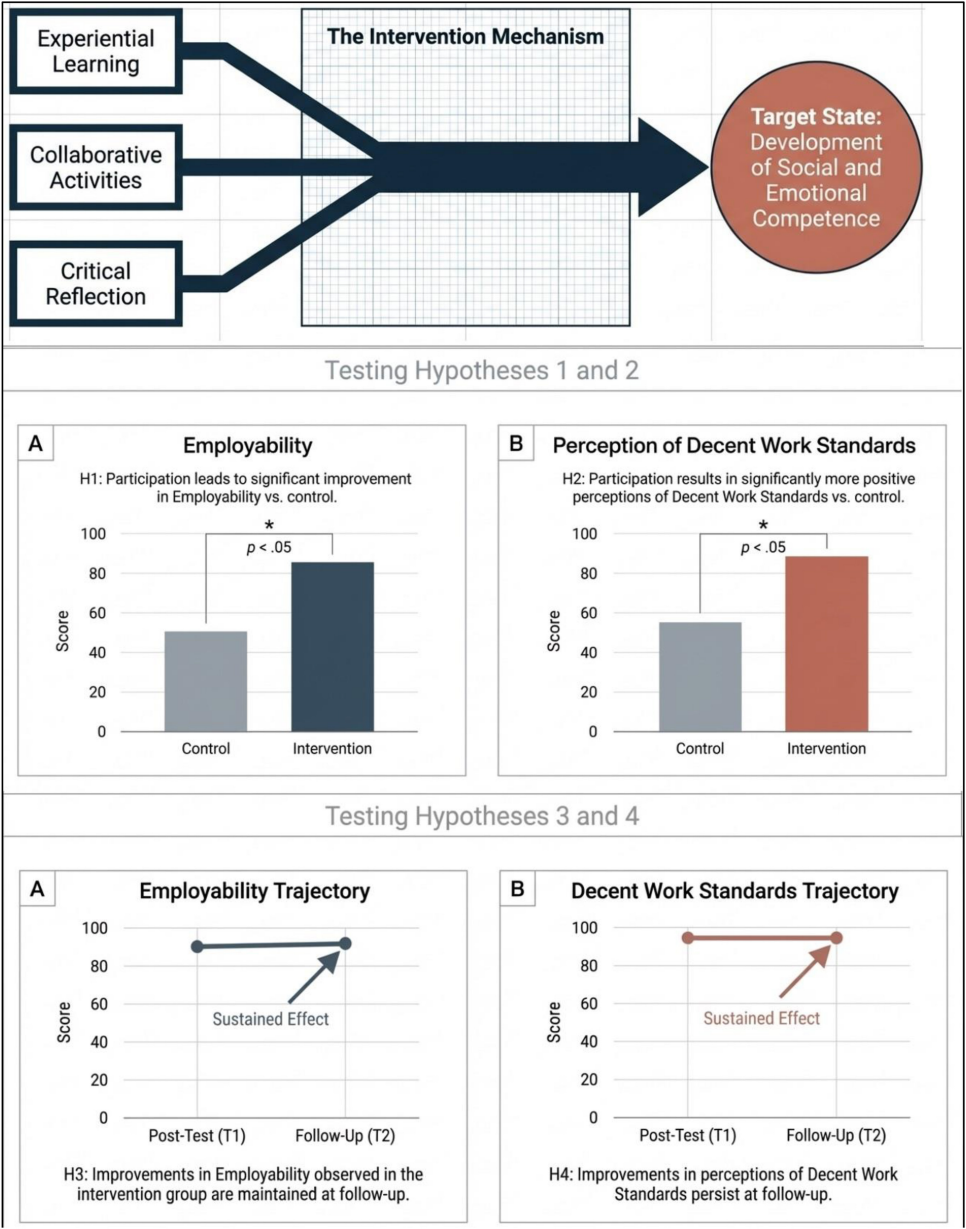
Conceptual model of the social and emotional competence–based intervention and tested hypotheses. **(A)** Employability results and **(B)** Decent work perception results. *p < 0.05

The within-subjects main effect of Time was statistically significant, *F*(1, 137) = 29.95, *p* < 0.001, with a partial eta squared (η^2^) of 0.179, indicating a moderate effect size (Table 2). Importantly, the Time × Group interaction was also statistically significant, *F*(1, 137) = 32.88, *p* < 0.001, η^2^ = 0.194, indicating that changes in employability over time differed significantly between the experimental and control groups (Figure 1).

**TABLE 2:** Results of repeated measures ANOVA for employability.

Source	SS	df	MS	*F*	*p*	η ^2^p
Time	4.68	1	4.68	29.95	< 0.001	0.179
Time × group	5.14	1	5.14	32.88	< 0.001	0.194
Error (time)	21.40	137	0.16			
Group (between)	4.90	1	4.90	6.32	0.013	0.044
Error (between)	106.20	137	0.78			

η^2^p, Partial eta squared; SS, Sum of Squares.

*Post-hoc* analyses of estimated marginal means showed that the control group exhibited no meaningful change in employability from pre-test to post-test (M_(_pre_)_ = 3.113, M_(_post_)_ = 3.100). In contrast, the experimental group demonstrated a substantial increase in employability following participation in the intervention (M_(_pre_)_ = 3.106, M_(_post_)_ = 3.642).

The assumption of sphericity was met (Mauchly’s W = 1.000), and all correction methods (Greenhouse–Geisser and Huynh–Feldt) yielded identical results. The between-subjects main effect of Group was also statistically significant, *F*(1, 137) = 6.32, *p* = 0.013, η^2^ = 0.044, indicating higher overall employability scores in the experimental group at post-test.

**FIGURE 2:**
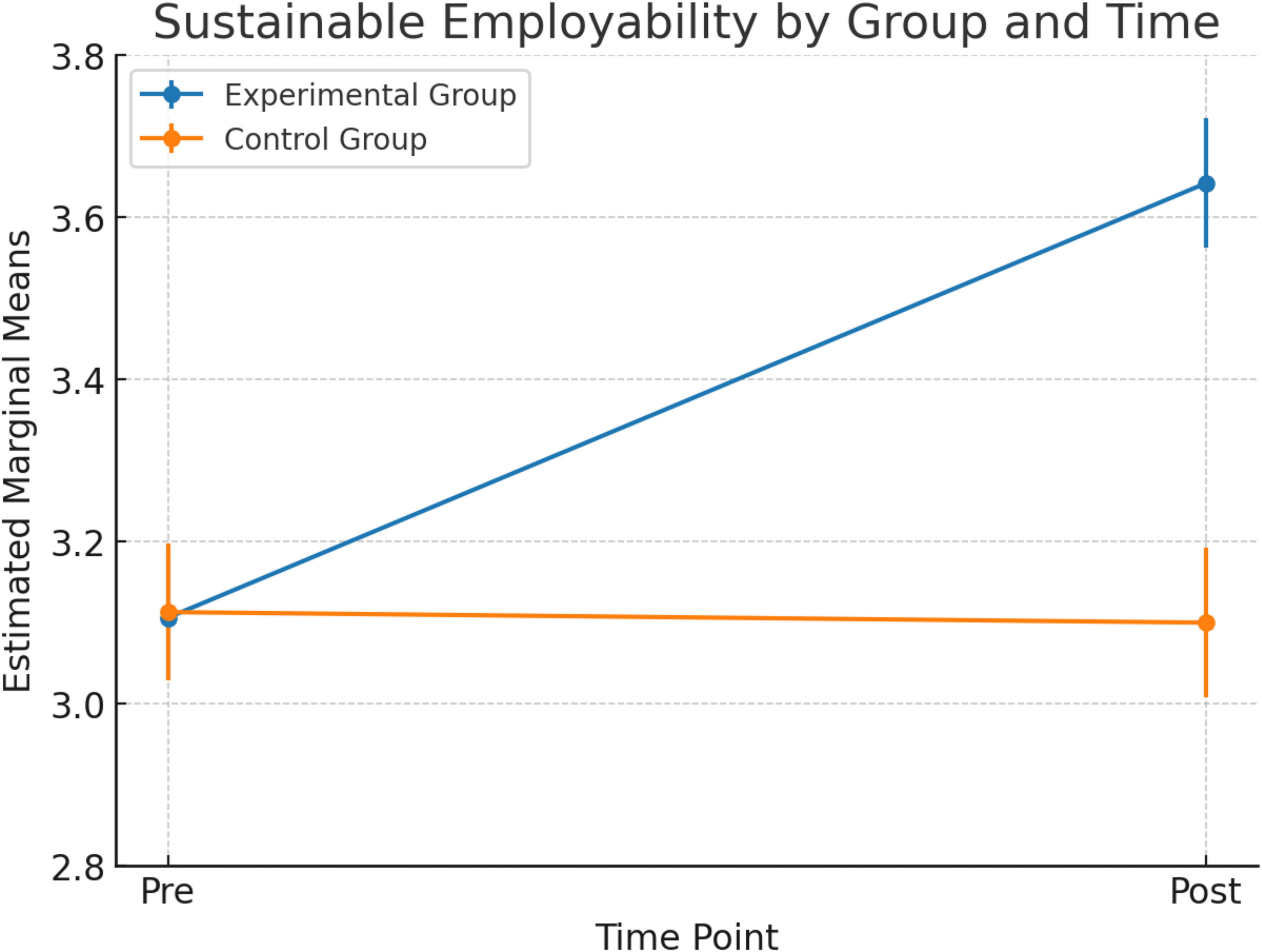
Line plot illustrating the estimated marginal means of employability at pre and post-intervention for both the experimental and control groups.

Figure 2 illustrates the estimated marginal means of employability at pre-test and post-test for the experimental and control groups. As shown, participants in the experimental group exhibited a marked increase in employability scores from pre-test to post-test, whereas employability scores in the control group remained largely stable across measurement occasions. Error bars represent standard errors. At post-test, the non-overlapping confidence intervals between the experimental and control groups visually correspond to the statistically significant Time × Group interaction reported in the mixed-design analysis.

### 4.3 Decent work standards

To examine the effects of the social and emotional competence–based intervention on perceptions of Decent Work Standards, a mixed-design repeated-measures ANOVA was conducted, with Time (pre-test vs. post-test) as a within-subjects factor and Group (experimental vs. control) as a between-subjects factor. The analysis included 139 participants, with 79 students in the experimental group and 60 students in the control group.

Descriptive statistics indicated that, at baseline, the experimental group reported a mean Decent Work score of *M* = 2.4759 (SD = 0.5321), whereas the control group reported a mean score of *M* = 2.5517 (SD = 0.6174). At post-test, perceptions of Decent Work increased in the experimental group (*M* = 2.9127, SD = 0.6089), while the control group showed only a slight change (*M* = 2.5900, SD = 0.8309).

The within-subjects main effect of Time was statistically significant, *F*(1, 137) = 20.95, *p* < 0.001, with a partial eta squared (η^2^) of 0.133, indicating a moderate time effect on Decent Work perceptions (Table 3). The Time × Group interaction was also statistically significant, *F*(1, 137) = 14.73, *p* < 0.001, η^2^ = 0.097, indicating that changes in Decent Work perceptions over time differed significantly between the experimental and control groups.

**TABLE 3:** Results of repeated measures ANOVA for decent work standards.

Source	SS	df	MS	*F*	*p*	η ^2^p
Time	3.85	1	3.85	20.95	< 0.001	0.133
Time × group	2.71	1	2.71	14.73	< 0.001	0.097
Error (time)	25.16	137	0.18			
Group (between)	1.04	1	1.04	1.60	0.208	0.012
Error (between)	89.07	137	0.65			

η^2^p = Partial eta squared; SS = Sum of Squares.

Analyses of estimated marginal means showed that the control group experienced only a marginal increase in Decent Work perceptions from pre-test to post-test (M? pre? = 2.552, M? post? = 2.590). In contrast, the experimental group demonstrated a more pronounced increase (M? pre? = 2.476, M? post? = 2.913).

The between-subjects main effect of Group was not statistically significant, *F*(1, 137) = 1.60, *p* = 0.208, η^2^ = 0.012, indicating no overall group differences when averaging across time points.

**FIGURE 3:**
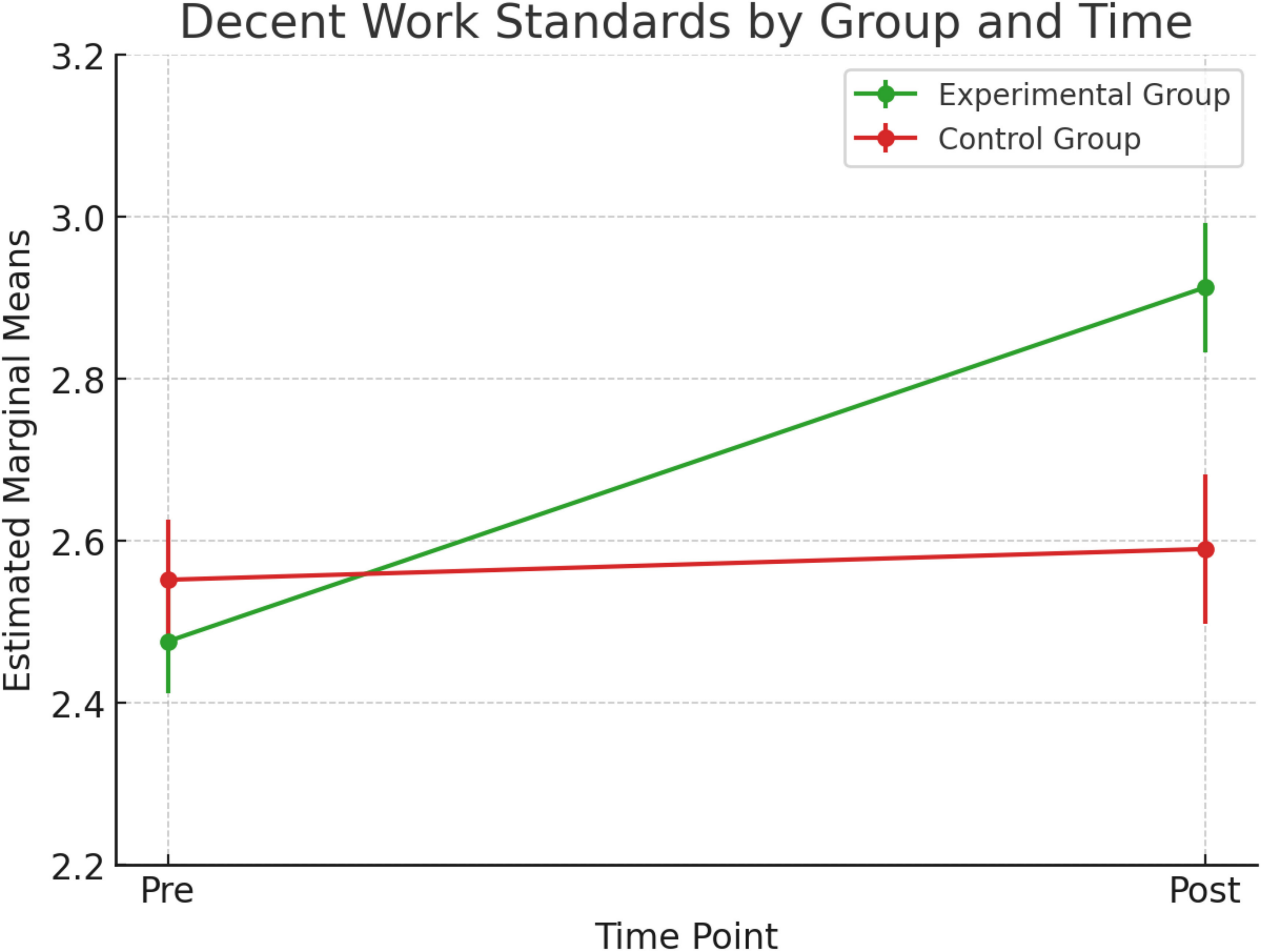
Line plot showing the estimated marginal means of decent work standards at pre and post-intervention for both the experimental and control groups.

Figure 3 presents the estimated marginal means of perceived Decent Work Standards for the experimental and control groups at pre-test and post-test. As shown, the experimental group exhibited an increase in Decent Work perceptions from pre-test to post-test, whereas the control group showed minimal change across measurement occasions. Error bars represent standard errors. At post-test, the divergence between the experimental and control groups, together with non-overlapping confidence intervals, visually corresponds to the statistically significant Time × Group interaction observed in the mixed-design analysis.

### 4.4 Maintenance of employability gains over time

To examine whether changes in employability observed at post-test were maintained over time, a repeated-measures ANOVA was conducted within the experimental group only (*n* = 79), with Time (pre-test, post-test, and follow-up) specified as the within-subjects factor.

Descriptive statistics indicated that employability increased from pre-test (*M* = 3.1060, SD = 0.6496) to post-test (*M* = 3.6424, SD = 0.5745) and remained at a comparable level at follow-up (*M* = 3.6345, SD = 0.4869).

The main effect of Time was statistically significant, *F*(2, 156) = 43.32, *p* < 0.001, with a large effect size (η^2^ = 0.357; Table 4). Mauchly’s test indicated that the assumption of sphericity was violated [W = 0.850, χ^2^(2) = 12.504, *p* = 0.002]; therefore, Greenhouse–Geisser corrections were applied. The corrected analysis confirmed the robustness of the Time effect, *F*(1.739, 135.67) = 43.32, *p* < 0.001, η^2^ = 0.357.

**TABLE 4:** Results of repeated measures ANOVA for employability over time (experimental group).

Source	SS	df	MS	*F*	*p*	η ^2^p
Time	14.93	2	7.47	43.32	<0.001	0.357
Linear contrast	11.03	1	11.03	47.48	<0.001	0.378
Quadratic contrast	3.90	1	3.90	34.71	<0.001	0.308
Error (time)	26.89	156	0.17			

η^2^p = Partial eta squared. The Greenhouse-Geisser correction was applied due to a violation of sphericity. SS, Sum of Squares.

Polynomial contrast analyses revealed a significant linear trend, *F*(1, 78) = 47.48, *p* < 0.001, η^2^ = 0.378, reflecting an increase in employability from pre-test to post-test. The quadratic trend was also statistically significant, *F*(1, 78) = 34.71, *p* < 0.001, η^2^ = 0.308, indicating that employability levels stabilized at follow-up rather than continuing to increase or decline.

### 4.5 Maintenance of improvements in decent work standards

To examine whether changes in perceptions of Decent Work Standards observed at post-test were maintained over time, a repeated-measures ANOVA was conducted within the experimental group only (*n* = 79), with Time (pre-test, post-test, and follow-up) specified as the within-subjects factor.

Descriptive statistics indicated that participants’ perceptions of Decent Work Standards increased from pre-test (*M* = 2.4759, SD = 0.5321) to post-test (*M* = 2.9127, SD = 0.6089) and remained relatively stable at follow-up (*M* = 2.8443, SD = 0.6242).

The main effect of Time was statistically significant, *F*(2, 156) = 28.67, *p* < 0.001, with a large effect size (η^2^ = 0.269; Table 5). Mauchly’s test indicated that the assumption of sphericity was violated (W = 0.590, *p* < 0.001); therefore, Greenhouse–Geisser corrections were applied. The corrected analysis confirmed the robustness of the Time effect, *F*(1.418, 110.63) = 28.67, *p* < 0.001, η^2^ = 0.269.

**TABLE 5:** Results of repeated measures ANOVA for decent work standards over time (experimental group).

Source	SS	df	MS	*F*	*p*	η ^2^p
Time	8.72	2	4.36	28.67	<0.001	0.269
Linear contrast	5.36	1	5.36	27.57	<0.001	0.261
Quadratic contrast	3.36	1	3.36	30.62	<0.001	0.282
Error (time)	23.72	156	0.15			

η^2^p = Partial eta squared. Greenhouse-Geisser correction was applied due to violation of sphericity. SS, Sum of Squares.

Polynomial contrast analyses revealed a significant linear trend, *F*(1, 78) = 27.57, *p* < 0.001, η^2^ = 0.261, reflecting an increase in Decent Work perceptions from pre-test to post-test. The quadratic trend was also statistically significant, *F*(1, 78) = 30.62, *p* < 0.001, η^2^ = 0.282, indicating a slight reduction in scores from post-test to follow-up, while remaining substantially above pre-test levels.

## 5 Discussion

The present study examined the effects of a social and emotional competence–based intervention on employability and perceptions of Decent Work Standards among students enrolled in Higher Vocational Education. Overall, the findings provide consistent support for the four hypotheses and contribute to theoretical discussions suggesting that socioemotional development may represent a key mechanism through which educational interventions can influence students’ preparedness for work and their expectations regarding employment quality.

Beyond the practical implications of the intervention, the findings also contribute to theoretical discussions on social and emotional competence in vocational education. First, the results support the view that socioemotional competencies constitute an important psychological resource shaping employability development prior to labor market entry. By demonstrating that a structured educational intervention can influence both employability perceptions and expectations regarding decent work, the study extends existing frameworks that link social and emotional competence with career development processes. Second, the findings provide empirical support for integrating perspectives from Self-Determination Theory and the Capability Approach in vocational education contexts, highlighting how socioemotional development can strengthen students’ perceived agency and capacity to pursue meaningful and dignified work. These findings are also consistent with a growing body of intervention research demonstrating that social and emotional learning programs can produce meaningful improvements in students’ socioemotional skills and broader developmental outcomes across educational contexts (Agırkan and Ergene, 2022; Durlak et al., 2011; Taylor et al., 2017). However, most previous intervention studies have focused primarily on behavioral adjustment, wellbeing, or academic outcomes, while relatively little research has examined how socioemotional competence development may shape students’ perceptions of employability and decent work prior to labor market entry. Compared to prior intervention studies in educational contexts, the present study extends existing research in several relevant ways. First, while many social and emotional learning interventions have been conducted in primary or general education settings, relatively few have focused specifically on Higher Vocational Education populations. Second, previous interventions have predominantly examined outcomes such as academic performance, behavioral adjustment, or general wellbeing, whereas the present study explicitly targets employability and perceptions of Decent Work Standards as central outcomes. Third, the use of a randomized controlled design combined with a longitudinal follow-up assessment strengthens the evidence base relative to studies relying on cross-sectional or short-term evaluation designs. Taken together, these distinctions position the present study as a complementary contribution that both aligns with and extends the current intervention literature in socioemotional development.

Consistent with Hypothesis 1, participation in the intervention was associated with significant improvements in employability among students in the experimental group. Importantly, these effects cannot be interpreted merely as gains in technical or task-related skills. Rather, they reflect broader improvements in socioemotional resources such as self-efficacy, emotional regulation, interpersonal competence, and career adaptability—capacities that are central components of social and emotional competence. This interpretation aligns with educational research demonstrating that socioemotional competencies play a critical role in students’ engagement, motivation, and adaptive functioning across educational contexts (Lee, 2024; Körkkö and Lutovac, 2024; Gebre et al., 2025). These results are broadly consistent with previous intervention studies indicating that programs designed to strengthen social and emotional competencies can enhance students’ career readiness, interpersonal functioning, and adaptive career behaviors (Dulas et al., 2025; Sáez-Delgado et al., 2025). Such findings suggest that socioemotional development may represent an important pathway through which educational interventions support students’ preparation for future employment.

From a socioemotional perspective, employability can be conceptualized as a dynamic psychological process rather than a static set of skills, emphasizing individuals’ capacity to regulate emotions, maintain motivation, and adapt to evolving work environments. The observed improvements are therefore consistent with evidence showing that interventions fostering emotional awareness, motivation, and reflective goal setting enhance students’ perceived ability to navigate future career challenges (Hassani, 2024; Tushar and Sooraksa, 2023). In vocational education contexts—where students often face early exposure to labor market uncertainty—such socioemotional resources may be particularly consequential for sustaining employability over time. Emerging research further suggests that interventions targeting emotional regulation, self-awareness, and goal-oriented reflection can strengthen students’ perceived capacity to manage career transitions and navigate uncertain labor market conditions (Lucas et al., 2025).

The findings also provide clear support for Hypothesis 2, indicating that students’ perceptions of Decent Work Standards improved following participation in the intervention. This result suggests that social and emotional competence development influences not only how students prepare for employment, but also how they conceptualize work quality, dignity, and fairness. Prior research grounded in the Psychology of Working Theory has emphasized that perceptions of decent work are shaped by individuals’ values, expectations, and sense of agency—processes that are inherently socioemotional (Duffy et al., 2017; Ma et al., 2023). While prior research grounded in the Psychology of Working Theory has primarily examined decent work perceptions among adults and working populations, the present findings extend this perspective by suggesting that expectations regarding fair and meaningful work may begin to develop during educational experiences that foster socioemotional reflection and agency. These effect sizes are broadly consistent with those reported in prior meta-analytic research on social and emotional learning interventions, where small-to-moderate effects are typically observed across educational outcomes. In this context, the magnitudes observed in the present study can be considered meaningful, particularly given the use of a randomized controlled design and the focus on vocational students, a population that has received comparatively less attention in intervention research.

The intervention’s emphasis on reflection, ethical discussion, and collaborative learning may have contributed to students’ enhanced awareness of decent work principles. This interpretation is also consistent with manipulation check responses, which indicated that participants particularly valued the reflective and collaborative components of the intervention, as well as their relevance for understanding work-related expectations. Similar findings have been reported in studies showing that socioemotional learning initiatives can shape students’ value orientations, wellbeing, and social inclusion (Frazier and Doyle Fosco, 2024; Sáez-Delgado et al., 2025). Rather than transmitting abstract policy concepts, the intervention provided experiential opportunities for students to engage emotionally and cognitively with questions of fairness, rights, and meaningful work. This interpretation is consistent with intervention research indicating that socioemotional learning initiatives can influence students’ attitudes, value orientations, and social awareness through experiential and reflective pedagogical approaches (Agırkan and Ergene, 2022).

Support for Hypotheses 3 and 4 further indicates that improvements in both employability and perceptions of Decent Work Standards were largely maintained over time. The longitudinal stability of these effects suggests that the intervention fostered relatively durable socioemotional changes rather than short-lived attitudinal shifts. This finding is consistent with research demonstrating that socioemotional competencies, once developed, can exert sustained influence on individuals’ functioning across contexts (Carpendale et al., 2025; Mehler et al., 2024). Similar patterns of sustained socioemotional development following educational interventions have been documented in previous research, suggesting that socioemotional competencies can remain relatively stable once internalized through structured learning experiences (Taylor et al., 2017).

The maintenance of employability gains may be explained by the intervention’s focus on transferable socioemotional capacities—such as problem solving, emotional regulation, and interpersonal communication—that remain relevant across changing work environments. Likewise, the persistence of improved perceptions of Decent Work Standards suggests that students internalized value-based frameworks for evaluating employment conditions, even in the absence of continued intervention exposure. These results align with evidence showing that socioemotional competence contributes to long-term wellbeing and adaptive functioning in educational and occupational settings (Galanis et al., 2024). Such patterns are consistent with findings indicating that socioemotional competencies function as transferable psychological resources that support adaptive functioning across educational and occupational contexts (Durlak et al., 2011).

Taken together, the findings underscore the importance of conceptualizing employability and decent work as outcomes shaped by both individual socioemotional development and educational context. Although much of the empirical literature on socioemotional learning interventions has been conducted in Western educational systems, recent studies suggest that similar mechanisms may operate across diverse cultural and institutional contexts. More broadly, the study contributes to emerging theoretical perspectives that position socioemotional competence as a foundational resource supporting individuals’ capacity to pursue meaningful and dignified work across different stages of the education-to-work transition. In the Chinese vocational education system—where students often encounter structural disadvantages and societal stigma—interventions that strengthen socioemotional competence may play a particularly important role in supporting positive transitions into work. Given that the sample included institutions from both economically developed regions (e.g., Suzhou) and less developed regions (e.g., Guizhou), regional disparities may also shape students’ expectations regarding what constitutes “decent work,” particularly in relation to job stability, income, and working conditions. Rather than positioning vocational education solely as a provider of technical labor, integrating socioemotional learning perspectives can enhance students’ agency, confidence, and expectations regarding meaningful employment. By examining these processes within Chinese Higher Vocational Education, the present study contributes to expanding the geographical scope of intervention research on socioemotional competence and employability development.

### 5.1 Limitations and directions for future research

While the present study provides robust empirical evidence for the effectiveness of a social and emotional competence–based intervention in enhancing employability and perceptions of Decent Work Standards among students in Higher Vocational Education in China, several methodological limitations should be acknowledged.

First, the sample was geographically restricted to students enrolled in vocational institutions within a limited regional context. Although vocational education systems in China share common structural features, regional differences in socioeconomic conditions, institutional resources, and labor market opportunities may influence both the implementation of socioemotional interventions and their outcomes. Future research should replicate and extend this study across diverse regions and institutional settings to strengthen the external validity of the findings.

Second, the study relied exclusively on self-report measures to assess employability and perceptions of Decent Work Standards. While the instruments employed demonstrated strong psychometric properties, self-reported data may be susceptible to response biases, including social desirability. Future studies would benefit from incorporating complementary objective or behavioral indicators—such as employment outcomes, job retention, supervisor evaluations, or observational measures of socioemotional functioning—to provide a more comprehensive assessment of intervention effects.

Third, although the study included a follow-up assessment approximately 4 months after the intervention, this time frame remains relatively short for evaluating the long-term sustainability of socioemotional development and employability-related outcomes. Longer longitudinal designs with multiple follow-up assessments are needed to examine whether gains in social and emotional competence, employability perceptions, and decent work expectations persist as students’ transition from education into actual employment contexts.

Fourth, the use of a waiting-list control group, while ethically appropriate, may have introduced expectancy or motivational effects that could influence participants’ responses. At the same time, this design may be considered a more conservative test of the intervention’s effectiveness, as any potential benefits derived from informational exposure in the control group would likely reduce between-group differences rather than inflate them. Future research should consider the inclusion of active control conditions offering alternative forms of engagement, which would allow for more precise isolation of the specific effects attributable to socioemotional competence development.

Fifth, although the study examined students’ perceptions of Decent Work Standards, it did not assess the correspondence between these perceptions and participants’ subsequent lived work experiences. Future investigations should explore how socioemotional competence developed during vocational education shapes the alignment between pre-employment expectations and actual employment conditions following graduation.

Finally, the intervention was implemented as a standardized program applied uniformly across participants. While this approach supports internal validity, it may not fully capture individual differences in students’ vocational pathways, baseline socioemotional competencies, or career aspirations. Future research should explore adaptive or differentiated intervention models that tailor socioemotional learning components to students’ specific needs, vocational fields, and developmental profiles.

Taken together, these limitations point to several directions for future research, including the use of more diverse samples, multimethod assessment strategies, extended longitudinal designs, active control conditions, examination of expectation–experience alignment in employment outcomes, and the development of flexible intervention models responsive to individual variability. Addressing these issues would contribute to a more nuanced understanding of how social and emotional competence can be cultivated within vocational education and how such development translates into employability and decent work outcomes.

### 5.2 Implications for higher vocational education curriculum development and occupational wellness

The findings of this study have several implications for curriculum development within Higher Vocational Education (HVE), particularly regarding the integration of social and emotional competence as a foundational resource for employability and informed perceptions of decent work.

First, the results highlight the importance of systematically embedding social and emotional competence development within vocational curricula, rather than addressing such competencies through isolated or optional training modules. Socioemotional capacities such as emotional regulation, interpersonal communication, collaborative problem-solving, self-efficacy, and adaptive coping have been consistently linked to employability, occupational functioning, and wellbeing in both educational and work contexts (Tong and Gao, 2022; Tushar and Sooraksa, 2023; Mehler et al., 2024). Integrating these competencies across disciplinary content allows for cumulative and developmentally appropriate learning, supporting students’ long-term adaptability and psychological preparedness for work.

Second, vocational curricula should explicitly incorporate reflective and value-oriented learning activities related to Decent Work Standards. The observed improvements in students’ perceptions of decent work suggest that socioemotional learning processes play a role in shaping how individuals conceptualize work quality, fairness, and dignity. Prior research grounded in the Psychology of Working framework indicates that perceptions of decent work are closely linked to values, expectations, and perceived agency—processes that are inherently socioemotional (Duffy et al., 2017; Ma et al., 2023; Roša et al., 2025). Embedding these themes within career development, ethics, or professional practice courses can help students develop realistic and psychologically grounded expectations regarding employment conditions.

Third, the findings underscore the pedagogical value of experiential and participatory learning approaches for fostering socioemotional development in vocational education. Approaches such as project-based learning, scenario-based discussions, collaborative problem-solving, and guided reflection have been shown to support the development of interpersonal competence, emotional awareness, and adaptive coping in educational settings (Frazier and Doyle Fosco, 2024; Hassani, 2024). In vocational contexts, these methods allow students to rehearse socioemotional responses to realistic work-related challenges, thereby strengthening the transferability of learning to future employment situations.

Fourth, curriculum design in HVE should recognize socioemotional competence as a core component of occupational wellbeing, rather than as an ancillary outcome. Research in both educational and occupational psychology suggests that socioemotional resources contribute to individuals’ capacity to manage workplace stressors, navigate interpersonal demands, and maintain psychological functioning across transitions (Galanis et al., 2024; Mehler et al., 2024). From this perspective, vocational education can play a preventive role by equipping students with socioemotional tools that support sustainable participation in work.

Finally, the results suggest that curriculum development efforts should align socioemotional learning objectives with broader institutional and societal goals related to sustainable employment and decent work. Integrating social and emotional competence within vocational curricula supports international frameworks emphasizing work quality, dignity, and wellbeing, including the International Labor Organization’s decent work agenda and Sustainable Development Goal 8 (Duffy et al., 2017; Roša et al., 2025). Such alignment reinforces the role of vocational education as a key context for fostering both employability and socially sustainable work trajectories.

## 6 Conclusion

This study provides robust empirical evidence supporting the effectiveness of a social and emotional competence–based intervention in enhancing employability and improving perceptions of Decent Work Standards among students enrolled in Higher Vocational Education programs in China. Participation in the intervention was associated with significant improvements in both outcomes, and these gains were largely maintained at follow-up, indicating that the effects extended beyond short-term attitudinal change.

By focusing on the development of socioemotional capacities—such as self-efficacy, emotional regulation, interpersonal competence, and reflective career orientation—the intervention addressed employability as a dynamic psychological process rather than as a narrow set of technical skills. The findings suggest that strengthening students’ social and emotional competence prior to labor market entry can enhance their perceived readiness to navigate work-related demands and uncertainties, as well as shape more informed and value-based expectations regarding employment quality.

Importantly, the observed improvements in perceptions of Decent Work Standards indicate that educational interventions can influence how students conceptualize work dignity, fairness, and sustainability before entering the workforce. This underscores the role of vocational education not only in preparing students for employment, but also in shaping their work-related values and expectations through socioemotional learning processes.

Taken together, the results highlight the relevance of pedagogical design and active student engagement in fostering durable educational outcomes. Interventions embedded within educational settings appear particularly well suited for supporting students’ socioemotional development during critical transitional phases, such as the shift from education to work. While the study was subject to several methodological limitations, the consistency and maintenance of the observed effects point to a promising and replicable educational approach.

Overall, this research contributes to the growing literature emphasizing social and emotional competence as a foundational resource for employability and decent work. By integrating socioemotional learning into vocational education, institutions may better support students’ psychological preparedness for work and promote more sustainable, meaningful transitions into employment in an evolving labor market.

## Data Availability

The datasets presented in this study can be found in online repositories. The names of the repository/repositories and accession number(s) can be found at: https://osf.io/vdafz/overview?view_only=f7a528fb331646e5a6b4826e1da3983b.
