# Supplementary material for: Developing social and emotional competence in higher vocational education: effects on employability and perceptions of decent work
Source: Front Psychol. 2026 May 29;17:1786673. doi: 10.3389/fpsyg.2026.1786673 (PMC13260081; doi:10.3389/fpsyg.2026.1786673)
Supplement: Supplementary file 1 [file Data_Sheet_1.pdf]

Appendix A. Social and Emotional Competence–Based Intervention Program

Program Title: Developing Social and Emotional Competence for Employability and Decent Work

Program Rationale: This intervention was designed to foster social and emotional competence among students enrolled in Higher Vocational Education programs. Drawing on social and emotional learning principles, Self-Determination Theory, and the Capability Approach, the program focused on developing socioemotional capacities relevant to students' transition into work, including self-efficacy, motivation, emotional regulation, interpersonal communication, resilience, reflective career planning, and awareness of decent work principles. Rather than targeting technical skills alone, the intervention emphasized experiential learning, critical reflection, and collaborative activities aimed at supporting students' psychological and social development in relation to future employment.

Total Duration: 6 sessions (1 session per week × 2 hours)

Target Group: Students enrolled in Higher Vocational Education programs

Facilitators: Educational psychologists and vocational instructors trained in participatory and socioemotional learning methodologies

Delivery Format: In-person, small-group workshops (15–20 students per group)

Structure and Content of the Intervention

Session	Core Theme	Targeted Social and Emotional Competencies	Objectives	Key Activities
1	Foundations of Social and Emotional Competence and Employability	Self-awareness; values clarification; future orientation	To introduce employability as a socioemotional process and support students in reflecting on personal values, aspirations, and long-term	Icebreakers; reflective journaling; vision boards; guided group discussions on labor market change

			career adaptability	
2	Self-Efficacy and Motivation for Work	Self-efficacy; intrinsic and extrinsic motivation; goal-setting	To strengthen students' confidence in their ability to pursue meaningful work and clarify motivational drivers related to employment	Self-assessment exercises; SMART goal setting; motivational mapping; peer feedback
3	Interpersonal and Emotional Competence in Work Contexts	Communication skills; collaboration; emotional regulation; problem-solving	To develop socioemotional skills necessary for effective interaction and cooperation in work-related settings	Role-playing scenarios; peer-led challenges; structured feedback
4	Socioemotional Understanding of Decent Work	Ethical awareness; perspective-taking; value-based judgment	To enhance students' understanding of decent work principles and their relevance for wellbeing, rights, and work values	Mini-lecture on the ILO framework; case studies; student-led discussions and presentations
5	Career Adaptability and Resilience	Emotional regulation; coping strategies; adaptive planning	To support students in developing flexible and realistic career plans that accommodate uncertainty, setbacks, and change	Career lifeline activity; stress management techniques; development of career portfolios

6	Commitment and Future-Oriented Action	Self-reflection; agency; commitment	To consolidate learning and encourage students to articulate personal commitments toward sustainable and decent work futures	Reflection circles; letters to the future self; individual action plans; peer pledges

Instructional Methods

Experiential Learning: Activities designed to engage students in cycles of experience, reflection, and application (e.g., simulations, journaling).

Collaborative Learning: Group-based tasks aimed at fostering interpersonal competence and shared responsibility.

Critical Reflection: Structured reflection connecting personal experiences with labor market realities, work values, and decent work principles.

Socratic Dialogue: Facilitated discussions encouraging ethical reasoning, perspective-taking, and critical examination of employment-related norms.
